# Eag Domains Regulate LQT Mutant hERG Channels in Human Induced Pluripotent Stem Cell-Derived Cardiomyocytes

**DOI:** 10.1371/journal.pone.0123951

**Published:** 2015-04-29

**Authors:** Qiang-ni Liu, Matthew C. Trudeau

**Affiliations:** Department of Physiology, University of Maryland School of Medicine, Baltimore, Maryland, United States of America; University of Tampere, FINLAND

## Abstract

Human Ether á go-go Related Gene potassium channels form the rapid component of the delayed-rectifier (I_Kr_) current in the heart. The N-terminal ‘eag’ domain, which is composed of a Per-Arnt-Sim (PAS) domain and a short PAS-cap region, is a critical regulator of hERG channel function. In previous studies, we showed that isolated eag (i-eag) domains rescued the dysfunction of long QT type-2 associated mutant hERG R56Q channels, by substituting for defective eag domains, when the channels were expressed in *Xenopus* oocytes or HEK 293 cells.Here, our goal was to determine whether the rescue of hERG R56Q channels by i-eag domains could be translated into the environment of cardiac myocytes. We expressed hERG R56Q channels in human induced pluripotent stem cell-derived cardiomyocytes (hiPSC-CMs) and measured electrical properties of the cells with whole-cell patch-clamp recordings. We found that, like in non-myocyte cells, hERG R56Q had defective, fast closing (deactivation) kinetics when expressed in hiPSC-CMs. We report here that i-eag domains slowed the deactivation kinetics of hERG R56Q channels in hiPSC-CMs. hERG R56Q channels prolonged the AP of hiPSCs, and the AP was shortened by co-expression of i-eag domains and hERG R56Q channels. We measured robust Förster Resonance Energy Transfer (FRET) between i-eag domains tagged with Cyan fluorescent protein (CFP) and hERG R56Q channels tagged with Citrine fluorescent proteins (Citrine), indicating their close proximity at the cell membrane in live iPSC-CMs. Together, functional regulation and FRET spectroscopy measurements indicated that i-eag domains interacted directly with hERG R56Q channels in hiPSC-CMs. These results mean that the regulatory role of i-eag domains is conserved in the cellular environment of human cardiomyocytes, indicating that i-eag domains may be useful as a biological therapeutic.

## Introduction

Human ether à go-go Related Gene K^+^ channels are members of the voltage-activated (K_v_) family of channels, which are characterized by opening in response to membrane depolarization and high selectivity for K^+^ ions. Like other K_v_ channels, hERG channels have six transmembrane domains and intracellular N- and C-terminal regions[1]. But, distinct from other K_v_ channels, the hERG N-terminal region contains an ‘eag’ domain which is comprised of a Per-Arnt-Sim (PAS) domain and an adjacent PAS-Cap region and a C-terminal region that contains a cyclic-nucleotide binding homology domain (CNBHD). The CNBHD is structurally similar to that of the CNG and HCN channels, but unlike those channels, the hERG CNBHD only weakly associates with cyclic nucleotides and does not undergo regulation by cyclic nucleotides[2]. In place of a cyclic nucleotide, the CNBHD in the hERG family of channels is bound by an intrinsic ligand, which is primarily composed of a three amino acid piece of the CNBHD itself[3–6].

hERG channels in the heart encode cardiac I_Kr_[7,8], a K^+^ current which helps to repolarize action potentials[9]. Mutations in hERG genes are associated with type 2 Long QT syndrome[10], a cardiac arrhythmia and predisposition to torsades de pointes tachycardias and sudden cardiac death. One of the unique properties of hERG K^+^ channels is their characteristic slow closing (deactivation), which requires the N-terminal eag domain[11–13] and the C-terminal CNBHD[14]. Slow deactivation in hERG is due to a mechanism in which the eag domain interacts directly with the CNBHD[14]. This interaction is also found in the closely–related mouse ether á go-go channels[15].

Mutations in the hERG channel eag domain accelerate deactivation kinetics and alter the steady-state inactivation properties of the channel[12,15–17]. One of the most profound mutations is an arginine to glutamine change at position 56 in the hERG eag domain, in which steady-state inactivation is right-shifted and channel deactivation is sped approximately 5-fold compared to that of wild-type hERG channels[15,16,17]. In previous studies, we showed that isolated eag (i-eag) domains interacted with some hERG channels with mutations in the eag domain, including hERG R56Q[17]. The i-eag domains rescued the dysfunction of hERG R56Q channels by replacing the defective eag domains when the channels were expressed in *Xenopus* oocytes or HEK 293 cells[17]. Here, we asked if the rescue of hERG R56Q channels by i-eag domains could be translated into the environment of cardiac myocytes. To answer this question, we overexpressed hERG R56Q channels in human induced pluripotent stem cell-derived cardiomyocytes (hiPSC-CMs) and measured electrical properties of the cells. Human iPSC-CMs have the advantage that they can be cultured for many weeks, which is sufficient for heterologous expression studies[18–21], making them an advantageous cell type for this study. We found that, like in non-myocyte cells, hERG R56Q had fast deactivation kinetics when expressed in hiPSC-CMs. We report here that i-eag domains slowed the deactivation kinetics in hERG R56Q currents in cardiomyocytes by making a direct association with hERG R56Q channels, as measured with Förster resonance energy transfer (FRET) spectroscopy, which means that the regulatory role of i-eag domains is maintained in the environment of cardiomyocytes. These results show that i-eag domains have the potential to act as biological therapeutics.

## Materials and Methods

### Mutagenesis

The hERG.Citrine, isolated eag domain and hERG(R56Q).Citrine constructs were previously described[17,22]. The adenoviral hERG.Citrine, hERG(R56Q).Citrine and i-eag.CFP were packaged by Vector Biolabs. Unless otherwise noted, all hERG constructs were fused to a fluorescent protein at the C-terminus.

### HEK293 cell expression and electrophysiology

HEK293 cells were cultured in Dulbecco’s Modified Eagle Medium (DMEM) supplemented with 10% fetal bovine serum, 1% L-glutamine, 1% penicillin and 1% streptomycin at 37°C and 5% CO_2_. When cells reached 50–70% confluence, they were infected by adenoviral wild-type hERG (WT hERG), hERG (R56Q) alone or hERG (R56Q) with i-eag. Cells were maintained in culture at 37°C in an adenovirus-only incubator. HEK293 cells were used to test the veracity of some of the adenovirally-delivered constructs. Whole-cell patch-clamp recordings from the HEK293 cells were conducted 24–48 hours after cells were plated in 35mM culture dishes and infected with adenovirus or transfected with cDNAs. Experiments were performed at room temperature. The external bath solution contained (in mM): 137 NaCl, 4 KCl, 1.8 CaCl_2_, 1 MgCl_2_, 10 Glucose, 10 HEPES, and 5 Tetraethylammonium (pH to 7.4 with NaOH), and the internal solution contained (in mM): 130 KCl, 1 MgCl_2_, 5 EGTA, 5 MgATP, and 10 HEPES (pH to 7.2 with KOH). Pipettes were pulled with a P-97 micropipette puller (Sutter Instruments, Novato, CA) with resistance between 2 and 5 MΩ when filled with the internal pipette solution. Whole-cell recordings were performed 24–48 hours post-transfection using an EPC-10 patch clamp amplifier (HEKA Instruments). Holding potential was -80 mV. Data were acquired using PatchMaster software, version 2.6 (HEKA Instruments), and analyzed using IgorPro Software, version 5.03 (Wavemetrics). The current-voltage (I-V) relationship was measured by plotting the peak current at the end of the depolarizing pulse normalized to the maximum peak tail current elicited by a step from -80 mV to 60 mV *versus* voltage. The voltage dependence of activation was measured by plotting the peak tail current versus voltage and fit with a Boltzmann function: y = 1/{1 + exp[(V_1/2_—V)/*k*]}, where V_1/2_ is the half-maximal activation potential and *k* is the slope factor. Steady-state inactivation was measured using a three-pulse protocol, as previously described[22]. Errors due to deactivation at -120 mV and -100 mV were corrected using the following equation: *I*_*corrected*_ = (g_difference_)(20-*E*_*rev*_); g_diference_ = (*I*_*peak*_—*I*_*end*_)/(*E*_*mem*_-*E*_*rev*_), where *I*_*peak*_ is the peak current during the 15 ms conditioning pulse, and *I*_*end*_ is the current at 15 ms. The resulting values were normalized, plotted versus voltage, and fit with a Boltzmann function. Deactivation current was fit with a double exponential function (y = A_1_ *e* ^-*t*/τ1^ + A_2_ *e* ^-*t*/τ2^), where *t* is time and τ is the time constant of deactivation. Current deactivation was fit with a double exponential function (y = A_1_e^-*t*/τ1^ + A_2_e^-*t*/τ2^), where *t* is time and τ is the time constant of deactivation.

### Human iPSC-CM culture

Human iPSC-CMs (iCell Cardiomyocytes) were purchased from Cellular Dynamics International (CDI, Madison, WI). For single cell patch-clamp recordings, glass coverslips were coated with 0.1% gelatin, placed into each well of a 12-well plates, and 2 mL of iCell Cardiomoycytes Plating Medium (iCPM) containing 40,000~60,000 cardiomyocytes were added to each coverslip. Cardiomyocytes at a low density were plated to permit culture of single cells and stored in an incubator maintained at 37°C and 5% CO_2_. After 48 hours, iCPM was replaced with cell culture media (iCell Cardiomyocytes Maintenance Medium, iCMM), which was exchanged every 48 hrs. Cardiomyocytes were maintained on coverslips for 4 to 21 days prior to use[23].

hERG currents and action potentials (APs) were recorded from single cardiomyocytes with perforated whole-cell patch-clamp techniques (using 240 μg/mL amphotericin B) at room temperature. For AP recordings with heterologous expression of WT hERG1a and hERG1a R56Q, cells were infected with the identical amount of adenovirus, cultured for the same period of time (24hrs) and AP recordings were performed on the following day and interleaved, as a control. For iPSC-CMs, APs were evoked by a current injection. The external bath solution contained (in mM): 140 NaCl, 5.4 KCl, 1.8 CaCl_2_, 1 MgCl_2_, 10 Glucose, and 10 HEPES (pH to 7.4 with NaOH), and the internal solution contained (in mM): 120 KCl, 1 MgCl_2_, 10 EGTA, 3 MgATP, and 10 HEPES (pH to 7.2 with KOH).

### FRET spectroscopy

To explore a direct interaction between adenoviral hERG R56Q and i-eag domains, we used Förster resonance energy transfer (FRET) spectroscopy. FRET spectroscopy directly tests protein-protein proximity when the distance between proteins is less than approximately 80Å. The FRET donor was i-eag fused to monomeric, enhanced CFP, and the FRET acceptor was hERG R56Q fused to Citrine. We measured FRET as stimulated emission of the acceptor by the donor (CFP). CFP and Citrine are FRET pairs and following excitation of the donor (CFP), energy is transferred from the donor to the acceptor. For both cell and spectroscopic imaging, two filter cubes (Chroma) we used a YFP cube and a custom CFP cube with a long-pass emission filter, as previously described[15]. Two spectrographic images were obtained from each cell, one with excitation at 436nm using the CFP cube, and the other with excitation at 500nm using the YFP cube. From these, the total emission spectrum and the Citrine emission spectrum were constructed, respectively. Emission spectra from cells transfected with CFP only, with excitation at 436nm, or with Citrine only, with excitation at 436nm and 500nm, were also measured as controls. To correct for bleed-through, the CFP spectrum measured from cells expressing donor only were subtracted from the total emission spectrum recorded with excitation at 436nm from cells expressing both donor and acceptor; this yielded a subtracted Citrine spectrum (F436_total_) free of donor contamination. F436_total_ contained two components—one due to direct excitation of Citrine at 436nm (F436_direct_) and one due to FRET (F436_FRET_). The F436_total_ spectrum was normalized to the Citrine emission spectrum with excitation at 500nm (F500) and was termed Ratio A: Ratio A = F436_total_/F500 = (F436_direct_/F500) + (F436_FRET_/F500). To solve for the F436_FRET_, the ratio of F436_direct_/F500, termed Ratio A_0_, was calculated from cells expressing acceptor (hERG-Citrine) only. We calculated the difference between Ratio A and Ratio A_0_ (from 530 to 560 nm) as Ratio A—Ratio A_0_ = F436_FRET_/F500, which is directly proportional to FRET efficiency.

### Statistics

The average data were expressed as mean ± S.E.M.. Statistical analysis was performed using unpaired Student’s *t*-test and one-way ANOVA. *p*<0.05 was considered as significant.

## Results

### Adenoviral i-eag domains rescue the gating-deficiencies of hERG R56Q channels expressed in HEK293 cells

**Fig 1:**
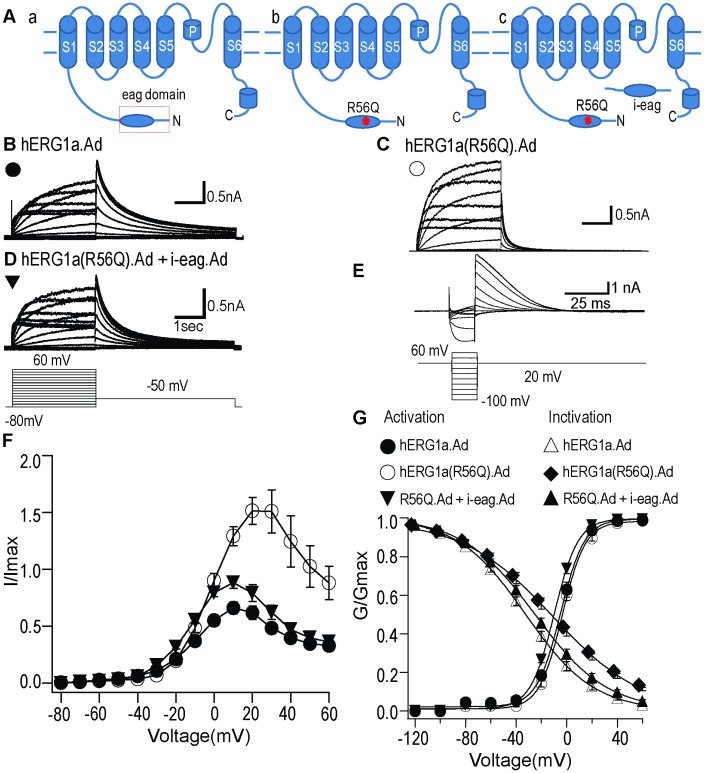
Adenoviral i-eag domains rescue the gating-deficiencies of hERG1a(R56Q) channels expressed in HEK293 cells. **A,** Schematic depicting hERG channel subunit: a, WT hERG1a; b, hERG1a(R56Q); c, hERG1a(R56Q) + i-eag. **B, C, D,** Representative current recordings from HEK293 cells expressing: WT hERG1a.Ad, hERG1a(R56Q).Ad, hERG1a(R56Q).Ad + i-eag.Ad. *Inset* represents the voltage protocol used. **E,** IV relationships of WT hERG1a, hERG1a(R56Q), and hERG1a(R56Q).Ad + i-eag.Ad. The current at the end of the depolarizing step was normalized to the maximum tail current and plotted versus voltage. **F,** Representative steady-state inactivation current recording from WT hERG1a (top) using a three-pulse protocol (bottom). **G,** Steady-state activation and inactivation curves. Steady-state activation curves were generated by normalizing tail currents at -50 mV to the maximum tail current and plotted *versus* voltage. Steady-state inactivation curves were generated by normalizing the peak current and plotted *versus* voltage. Both steady-state activation and inactivation curves were fit with a Boltzmann function. All values were plotted as mean ± S.E.M; n = 5–13 cells.

In this study, our goal was to test whether the genetically encoded, isolated eag (i-eag) domains could rescue the function of a hERG LQTS mutant channel in the environment of cardiac myocytes. We chose to use hiPSC-CMs because they are human cells, can be cultured for several weeks (allowing for the expression of recombinant genes) and have some properties of cardiomyocytes, such as APs. We found that transfection of hERG channels in mammalian expression vectors (e.g. pcDNA3) was not effective for hiPSC-CMs, so to carry out these experiments, we first performed a positive control by testing the expression of adenovirally delivered wild-type hERG, hERG(R56Q) and hERG(R56Q) + i-eag domains (Fig 1A) in HEK293 cells.

Whole-cell patch-clamp recordings showed robust expression of each hERG channel (Fig 1B, 1C and 1D) and normalized current-voltage (I-V) curves showed that hERG R56Q had significantly larger current amplitudes relative to that of WT hERG (Fig 1F). Co-expression with i-eag domains changed the I-V relationship of hERG R56Q channels to be more similar to that of hERG channels (Fig 1F). Steady-state activation curves were generated from tail currents, normalized to the maximum tail current and fit with a Boltzmann function. We found that hERG R56Q channels did not change the activation kinetics of WT hERG channels (Fig 1G, Table 1). We analyzed the tail current produced by a step to 60 mV from a potential of -80 mV and the decay was fit with a double exponential function to derive fast and slow time constants of deactivation. hERG R56Q currents had a faster time course of decay (deactivation) compared with that of WT hERG at -50 mV and deactivation time course was partially restored by i-eag domains (Fig 1B, 1C and 1D; Table 2). We also measured the voltage-dependent steady-state inactivation of hERG R56Q and the subsequent rescue by i-eag domains. To analyze the steady-state inactivation kinetics, a three-pulse protocol was used (Fig 1E). After a long depolarizing pulse to maximally activate the channels, the membrane potential was stepped to various potential ranging from -120 mV to 60 mV in increments of 20 mV for 15 ms to allow the channels to recover from inactivation and reach a steady-state level. The resulting peak outward currents were measured at +20 mV. The inactivating outward currents (measured at + 20 mV) were normalized and plotted against the voltage and fit with Boltzmann function, giving the steady-state inactivation curve (Fig 1G). The steady-state inactivation curve was positively shifted by hERG R56Q, with a V_1/2_ of -13.6 mV compared with -32.2 mV for WT hERG (*p*<0.05). Coexpression with adenoviral i-eag domains restored the steady-state inactivation curve (Fig 1G) with a V_1/2_ of -24.7 mV, which was not different from that of WT hERG channels (Table 1). Our data showed that the adenoviral delivery of i-eag domains resulted in i-eag domain regulation that was similar to transfection of cDNA or injection of RNA as reported previously[17,22]

**Table 1:** Steady-state activation properties for adenoviral hERG1a, hERG1a(R56Q) and hERG1a(R56Q)+ i-eag.Ad in HEK293 cells and hiPSC-CMs.

Groups	HEK293 cells		Human iPSC-CM		n
	*V*_*1/2*_	*k*	*V*_*1/2*_	*k*	
hERG1a.Ad	-4.44 ± 1.53	8.55 ± 0.33	6.50 ± 1.00	6.16 ± 0.57	13–16
hERG1a(R56Q).Ad	-3.34 ± 1.07	9.00 ± 0.53	3.12 ± 1.08	6.92 ± 0.23	13–18
hERG1a(R56Q).Ad+ i-eag.Ad	-9.23 ± 0.86	9.23 ± 0.63	3.84 ± 1.04	7.01 ± 0.24	18

**Table 2:** Deactivation time constants of hERG currents in HEK293 cells and hiPSC-CMs.

Groups	voltage	HEK293 cell		Human iPSC-CM	
	(mV)	τ_fast_(ms)	τ_slow_(ms)	τ_fast_(ms)	τ_slow_(ms)
hERG1a.Ad	-120	50.1 ± 4.5	796.2 ± 92.0	29.6 ± 5.0	39.7 ± 6.0
	-100	97.4 ± 8.9	1043.5 ± 89.0	53.0 ± 10.1	118.6 ± 23.4
	-80	166.5 ± 45.7	562.9 ± 58.9	145.8 ± 31.6	1587.5 ± 325.0
	-60	224.4 ± 10.7	1125.8 ± 110.2	236.8 ± 21.6	1428.9 ± 157.7
	-40	303.6 ± 19.5	1878.2 ± 109.4	329.4 ± 30.7	2399.7 ± 129.8
	-20	477.4 ± 59.2	2328.2 ± 232.8	1651.5 ± 333.2	2148.6 ± 189.5
hERG1a (R56Q).Ad	-120	14.8 ± 3.7*	218.3 ± 58.1*	17.3 ± 2.3	23.4 ± 3.7
	-100	22.6 ± 4.9*	269.8 ± 67.4*	21.1 ± 2.3*	29.3 ± 5.1*
	-80	29.2 ± 2.5*	215.3 ± 26.0*	31.9 ± 3.3*	96.2 ± 28.9*
	-60	56.2 ± 6.3*	286.3 ± 28.8*	54.2 ± 3.9*	256.7 ± 25.0*
	-40	90.7 ± 4.9*	567.6 ± 60.6*	96.6 ± 7.0*	602.6 ± 42.8*
	-20	149.1 ± 8.4*	1029.9 ± 63.8*	196.1 ± 15.9*	1268.1 ± 72.2*
hERG1a(R56Q).Ad + i-eag.Ad	-120	25.1 ± 3.4	339.1 ± 89.7	29.8 ± 5.3	28.7 ± 4.2
	-100	41.4 ± 5.8^#^	344.3 ± 68.4	43.2 ± 7.7^#^	49.2 ± 7.3^#^
	-80	48.5 ± 14.4	263.4 ± 28.7	70.0 ± 10.1^#^	306.1 ± 83.3^#^
	-60	106.6 ± 11.8^#^	421.2 ± 38.1^#^	132.6 ± 14.6^#^	652.5 ± 87.9^#^
	-40	183.9 ± 22.2^#^	996.4 ± 108.9^#^	243.5 ± 23.2^#^	1530.2 ± 96.8^#^
	-20	306.9 ± 42.4^#^	1782.8 ± 296.3^#^	326.3 ± 20.4^#^	2339.1 ± 132.6^#^

*p<0.05 vs. hERG1a.Ad;

^#^p <0.05 vs. hERG1a (R56Q).Ad. Number of cells for each group is between 6~14, 5~19 and 6~18, respectively.

### Adenoviral i-eag domains restore slow deactivation in hERG R56Q channels in HEK293 cells

**Fig 2:**
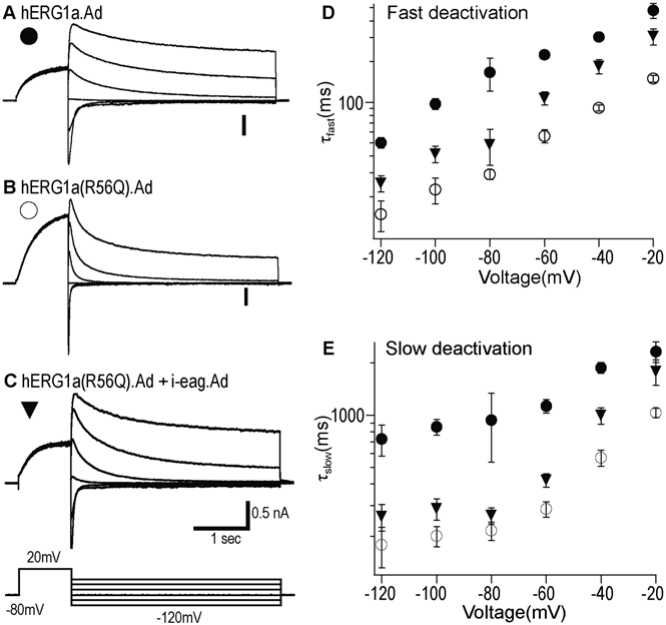
Adenoviral i-eag domains restore slow deactivation in HEK293 cells. Representative tail current recordings from HEK293 cells expressing: **A,** WT hERG1a.Ad; **B,** hERG1a(R56Q).Ad; **C,** hERG1a(R56Q).Ad + i-eag.Ad. *Inset* represents the voltage protocol used. **D,** Tail currents were fit with a double exponential function and the mean τ_fast_ (top) and τ_slow_ (bottom) values were plotted against voltage on a logarithmic scale. All values were plotted as mean ± S.E.M; n = 6–8 cells.

To characterize the altered deactivation for hERG R56Q channel and examine the rescue by adenoviral i-eag domains in more detail, we used a voltage command protocol to elicit a series of deactivating current traces. Following channel activation, tail currents were produced by a series of steps ranging from -120 mV to -20 mV in 20 mV increments, and then were fit with a double exponential function to derive fast and slow deactivation time constants. Compared with WT hERG.Ad (Fig 2A), hERG(R56Q).Ad (Fig 2B) exhibited accelerated deactivation kinetics, while coexpression of hERG(R56Q).Ad with adenoviral i-eag domains (Fig 2C) partially restored slow deactivation (Fig 2D and 2E; Table 2). Therefore, the WT hERG, hERG(R56Q) and i-eag domain expressed using adenovirus had similar properties as those expressed using the pcDNA3 vector in HEK293 cells or RNAs in *Xenopus* oocytes.

### Aberrant hERG R56Q current generated by an action potential command was rescued by i-eag domains

**Fig 3:**
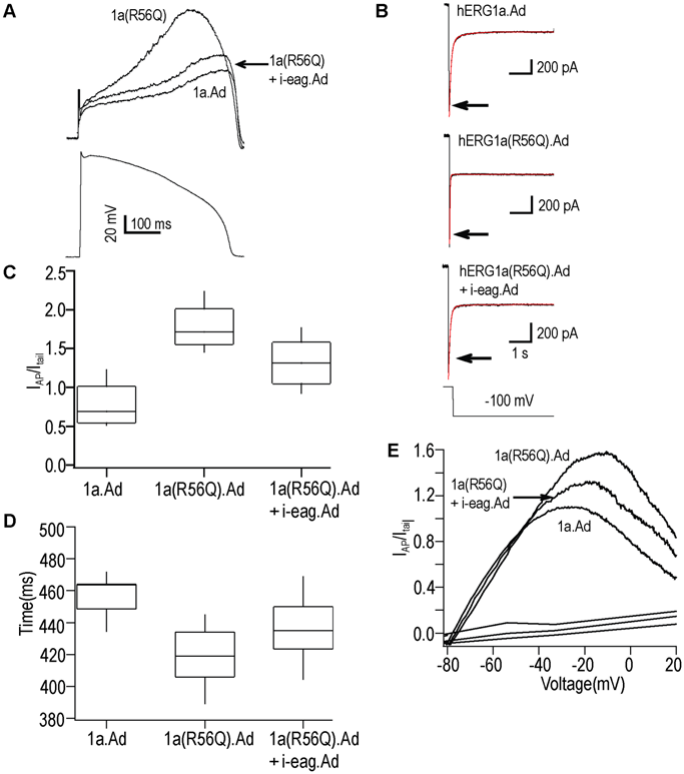
Adenoviral i-eag domains regulated the hERG R56Q current recorded with an AP waveform command. Representative current recordings: **A** (top) from WT hERG1a (black), hERG1a R56Q (red), and hERG R56Q + i-eag (blue) elicited with a voltage command mimicking a ventricular action potential **A** (bottom). **B**, Tail currents were generated by a step to −100 from 60 mV from same cells as in A). Double exponential fits (red traces) were extrapolated back to the moment of voltage change to obtain the peak tail current value (arrow). **C**, Box plot of peak currents elicited with AP command voltage (as in A) were normalized to peak tail current (as in B) to normalize for variations in channel expression between cells. **D**, Box plot of the time of the peak current elicited with the AP command voltage. For box plots, the *middle line* is the mean, the *top* and *bottom lines* are the 75th and 25th percentiles, respectively, and the *s*traight lines are the 90th and 10th percentiles. n = 6–9 cells.**E**, Plot of currents in A) versus AP command voltage.

To test the role of i-eag domains on hERG R56Q channels under voltage clamp conditions that mimic physiological conditions, we used a voltage command (Fig 3) that mimics a ventricular action potential (AP). Currents elicited using the AP command (Fig 3A) were normalized to the extrapolated peak tail current at −100 mV from the same cell (Fig 3B) for ease of comparison. Representative current recordings (Fig 3A) showed that hERG R56Q channels had a larger outward current that peaked earlier compared to that for WT hERG (Fig 3A, 3C, and 3D, p <0.01), but that the hERG1a R56Q current during repolarization (from approximately -50 to -80 mV) is smaller than that of the WT hERG1a current (Fig 3A and 3E), supporting the results of previous studies[16,22]. We next co-expressed i-eag with hERG R56Q and we determined that the peak outward current and time to peak were shifted toward the values for WT hERG (Fig 3A, 3C, and 3D, p >0.01) and that the current during repolarization (from approximately -50 to -80 mV) was larger than that of hERG1a R56Q (Fig 3A and 3E). These findings demonstrate that i-eag.Ad partially restored WT-like current properties to gating-deficient hERG R56Q channels in response to a voltage command that mimics a ventricular action potential waveform.

### Adenoviral i-eag domains rescued the gating-deficiencies of hERG R56Q channels expressed in hiPSC-CMs

**Fig 4:**
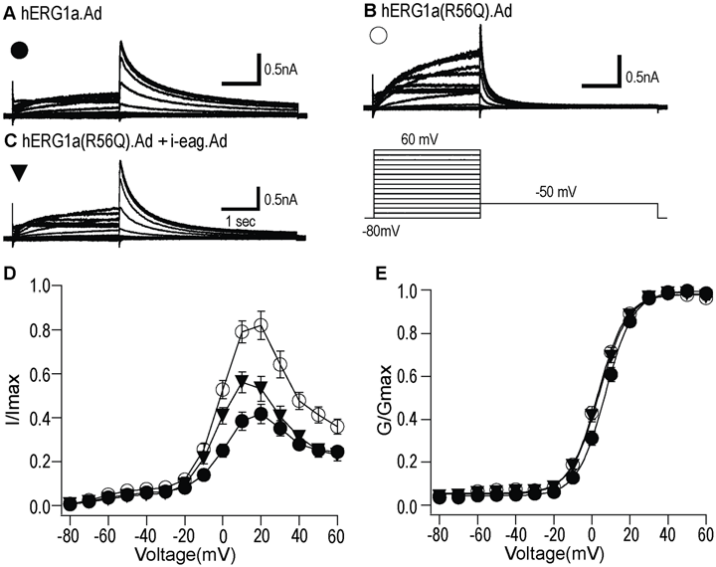
Adenoviral i-eag domains rescued the gating-deficiencies of hERG1a(R56Q) channels expressed in hiPSC-CMs. Voltage-clamp recordings of hERG currents measured from hiPSC-CMs infected by: **A,** WT hERG1a.Ad; **B,** hERG1a(R56Q).Ad; **C,** hERG1a(R56Q).Ad + i-eag.Ad. *Inset* represents the protocol used. **D,** I-V relationships for WT hERG1a.Ad, hERG1a(R56Q).Ad and hERG1a(R56Q).Ad + i-eag.Ad. The current at the end of depolarizing step was normalized to the maximum tail current and plotted versus voltage. All values were plotted as mean ± S.E.M; n> = 15 cells.

Here, to address the properties of the hERG R56Q mutation and i-eag domains in the environment of cardiomyocytes, we infected hiPSC-CMs with adenoviral hERG genes. We heterologously expressed WT hERG.Ad, hERG(R56Q).Ad and hERG(R56Q).Ad + i-eag.Ad in hiPSC-CMs and measured robust currents using the perforated whole-cell patch-clamp method (Fig 4A, 4B and 4C). We found that normalized current-voltage (I-V) curves (Fig 4D) for hERG(R56Q).Ad had significantly larger current amplitudes compared with that of WT hERG.Ad and the difference was similar to that in HEK293 cells (see Fig 1). Adenoviral i- eag domains reduced the normalized I-V curve of hERG R56Q channels (Fig 4D), similar to what we found in HEK293 cells (Fig 1). There were no significant differences in steady-state activation curves (Fig 4E, Table 1). To examine deactivation gating, we fit tail currents at -50 mV (Fig 4A, 4B and 4C) with a double exponential function and found that hERG(R56Q).Ad channels had a faster rate of deactivation compared with that of WT hERG.Ad, and that co-expressing i-eag domains with hERG(R56Q) slowed deactivation (Table 1), indicating that hERG(R56Q).Ad retained its aberrant gating properties in cardiomyocytes. These results show that adenoviral delivery of i-eag domains restored the I-V relationship and the deactivation properties of hERG R56Q channels expressed in hiPSC-CMs.

### Adenoviral i-eag domains regulated hERG R56Q channels deactivation in hiPSC-CMs

**Fig 5:**
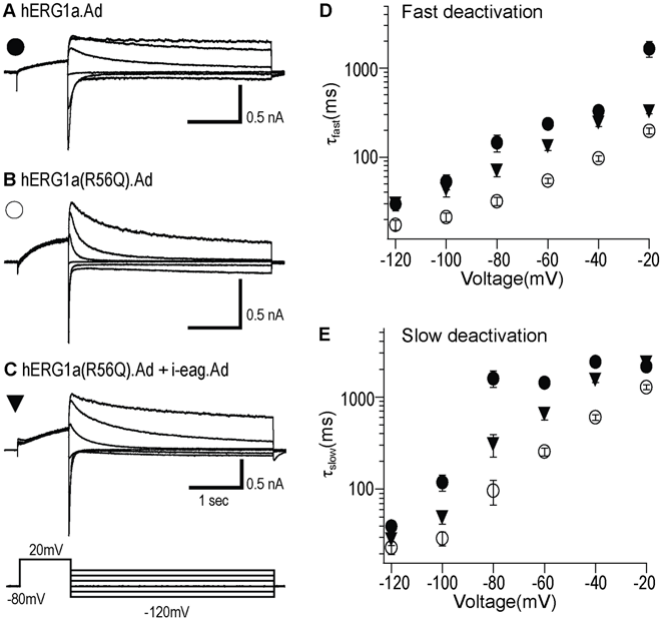
Adenoviral i-eag domains restored slow deactivation in hiPSC-CMs. Representative tail currents recorded from hiPSC-CMs infected by: **A,** WT hERG1a.Ad; **B,** hERG1a(R56Q).Ad; **C,** hERG1a(R56Q).Ad + i-eag.Ad. *Inset* represents the voltage protocol used. **D,** Tails were fit with a double exponential function, and mean τ_fast_ (top) and τ_slow_ (bottom) values were plotted against voltage on a logarithmic scale. All values were plotted as mean ± S.E.M.; n> = 15 cells.

We characterized the altered deactivation of hERG R56Q channels and examined the rescue of adenoviral i-eag domains in more detail, using a voltage command protocol to elicit a series of deactivating traces. Compared with that of hERG.Ad, hERG(R56Q).Ad channels exhibited accelerated deactivation kinetics (Fig 5A, 5B, 5D and 5E). Co-expression of adenoviral i-eag restored the slow deactivation in hERG R56Q channels over a range of voltages (Fig 5C, 5D and 5E; Table 2). These data suggested that hERG R56Q defective channel gating (faster deactivation) was maintained in hiPSC-CMs, as in HEK293 cells and oocytes, and the adenoviral i-eag domains rescued the aberrant deactivation kinetics.

### Heterologously expressed hERG currents were isolated as E-4031 sensitive currents

**Fig 6:**
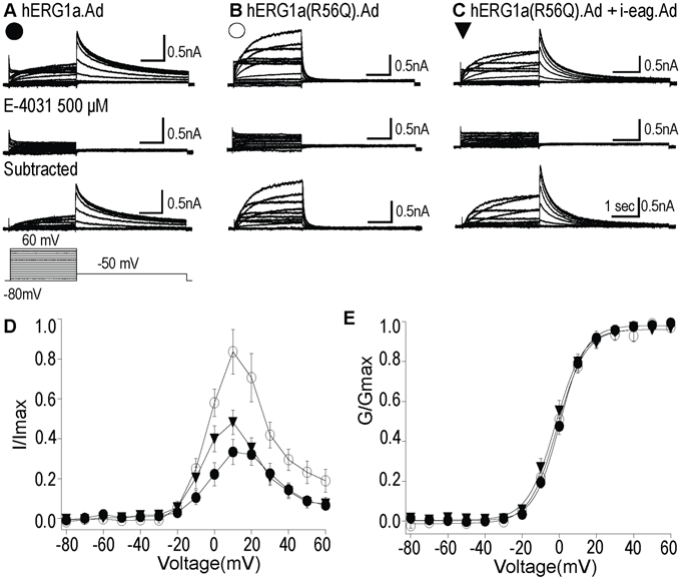
hERG currents were isolated as E-4031 sensitive current. hERG currents overexpressed in hiPSC-CMs were isolated as E-4031-sensitive currents. **A,B,C,** The data showed families of current traces for WT hERG1a.Ad, hERG1a(R56Q).Ad and hERG1a(R56Q) + i-eag.Ad (top), after the addition of E4031(500 μM; Middle), and digitally subtracted traces to give the E-4031 sensitive currents (bottom). **D,** I-V relationships for each hERG channel above. The current at the end of depolarizing step was normalized to the maximum tail current and plotted versus voltage. **E,** Steady-state activation curves for each hERG channel above. All values were plotted as mean ± S.E.M.; n> = 15 cells.

Since hiPSC-CMs likely contain other voltage-activated ionic currents, we investigated the hERG component of the voltage-activated currents, by isolating hERG current as an E-4031-sensitive current. Representative current traces of WT hERG.Ad (Fig 6A), hERG(R56Q).Ad (Fig 6B), and hERG(R56Q).Ad + i-eag.Ad (Fig 6C) in control conditions (top), after the addition of E-4031 (500 μM; middle), and as digitally subtracted traces to give the E-4031-sensitive current (bottom). Data with E-4031 were collected 5 min after drug application. The peak tail current densities for WT hERG.Ad, hERG(R56Q).Ad and hERG(R56Q).Ad + i-eag.Ad were (58.8 ± 6.38) pA/pF, (51.6 ± 9.39) pA/pF and (47.0 ± 6.20) pA/pF, respectively. The results indicate that the majority of the measured K^+^ current were from heterologously expressed hERG channels. The I-V relationship shows less rectification for hERG R56Q channels than for WT hERG channels and rectification was restored when hERG R56Q was co-expressed with i-eag domains (Fig 6D). There was no measurable change between the G-V curves for the three different hERG currents (Fig 6E, Table 1).

### FRET between i-eag domains and hERG R56Q channels in hiPSC-CMs

**Fig 7:**
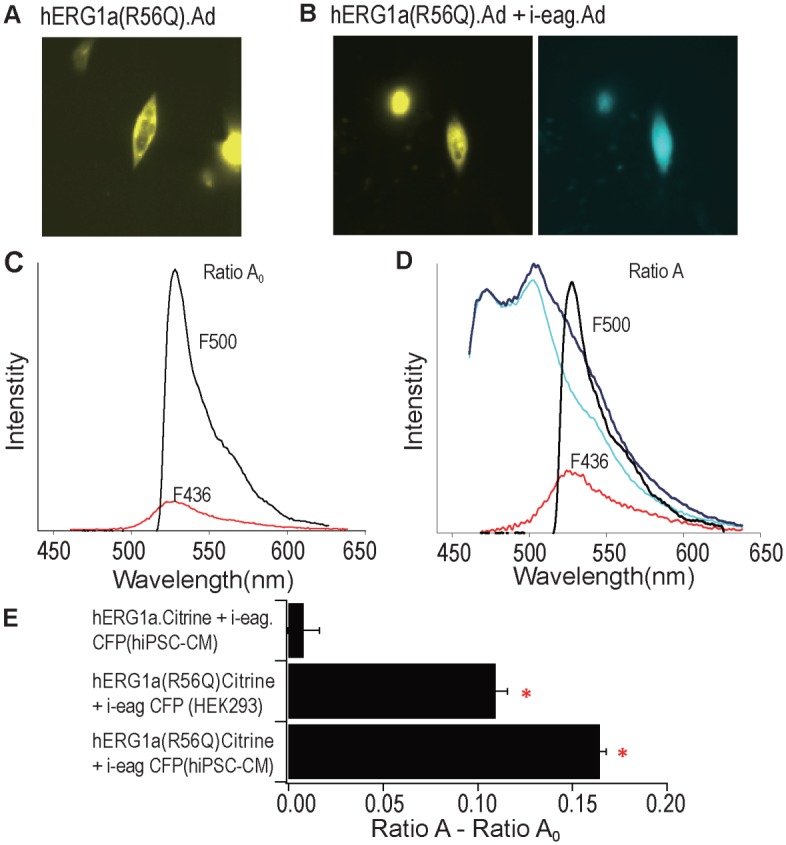
FRET between i-eag domains and hERG R56Q channels in hiPSCs. Images of cells: **A,** hERG1a(R56Q).Citrine.Ad; **B,** hERG1a(R56Q).Citrine.Ad + i-eag.CFP.Ad. **C,** Determination of Ratio A_0_. The emission spectra from hiPSCs expressing hERG1a(R56Q).Citrine was determined with excitation at 436 nm and 500 nm. Ratio A_0_ is the F436 spectra normalized to F500 spectra. **D,** Spectra method for measuring FRET and determining Ratio A. Spectra (dark blue trace) was measured from cells coexpressing i-eag.CFP.Ad + hERG1a(R56Q).Citrine.Ad by excitation at 436nm. Emission spectra of CFP (cyan) were measured in a control experiment from hiPSCs expressing i-eag.CFP. Extracted spectra (F436, red trace) is the cyan spectra subtracted from the dark blue trace and contains the emission of Citrine. Spectrum (F500, black trace) was measured from excitation of Citrine at 500 nm. Ratio A is the F436 spectra normalized to F500 spectra. **E,** Bar graph of Ratio A- Ratio A_0_, a value directly related to FRET efficiency. n = 7–9 cells. *p<0.05 for FRET between hERG1a(R56Q) and i-eag-CFP domains compared to hERG1a and i-eag-CFP control.

To test the physical proximity of i-eag domains and hERG R56Q channels, we performed FRET experiments. FRET between two proteins indicates that they are within close (less than 80 Å) proximity to one another. We expressed hERG(R56Q).Citrine alone (Fig 7A and 7C) and with i-eag-CFP domains (Fig 7B and 7D) and measured fluorescence intensities. Using spectral FRET, we found robust FRET between hERG(R56Q).Citrine and i-eag.CFP domains (Fig 7E). As a positive control, we detected FRET between hERG(R56Q).Citrine and i-eag.CFP in HEK293 cells (Fig 7E). In negative control experiments, we did not detect FRET between WT-hERG.Citrine and i-eag.CFP domains (Fig 7E). These FRET results, together with the functional regulation of hERG R56Q by i-eag domains, strongly suggest that eag domains make a direct interaction with hERG R56Q channels to regulate and restore deactivation gating in hiPSC-CMs. Since eag domains have FRET with hERG R56Q channels, but not WT hERG channels, we propose the R56Q mutation weakens the known eag domain interaction with the CNBHD[4,14,15] and that this weakened interaction underlies LQT2.

### i-eag domains regulate excitable properties in hiPSC-CMs

**Fig 8:**
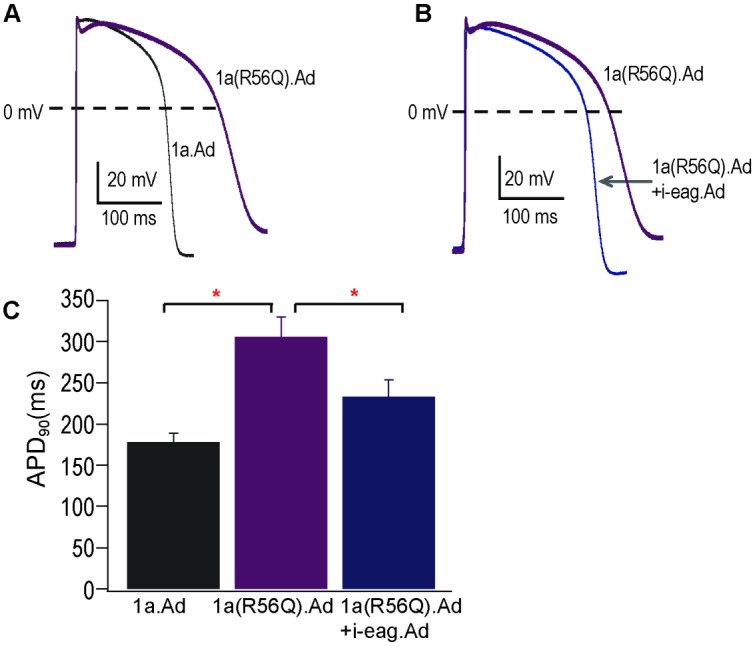
i-eag domains regulate action potentials. Current clamp recording of action potentials from hiPSC-CMs expressing WT hERG1a.Ad, hERG1a(R56Q).Ad and hERG1a(R56Q).Ad + i-eag.Ad. **A,** hERG1a(R56Q) increased the action potential duration (*p <0.05 compared to WT hERG1a.Ad). **B,** Coexpression of i-eag.Ad decreased the action potential duration in cells with hERG1a(R56Q) (*p <0.05 compared to hERG1a(R56Q).Ad). **C,** Histogram of APD_90_ values in hiPSC-CMs infected by WT hERG1a.Ad, hERG1a(R56Q).Ad, and hERG1a(R56Q).Ad + i-eag.Ad. n = 12 for each. Scale bar is 50 s and 20 mV.

We next examined the effects of hERG channels on action potentials of hiPSC-CMs (Fig 8). Compared to the AP from positive control cells expressing WT hERG1a channels, the AP from cells expressing hERG1a R56Q had a longer duration (Fig 8A and 8C). Compared to the AP from cells expressing hERG1a R56Q, cells co-expressing i-eag domains and hERG1a R56Q had APs that were of shorter duration (Fig 8B and 8C). We interpret these results to mean that i-eag domains regulated hERG1a R56Q channels and shortened the APs.

## Discussion

The primary goal of these studies was to determine whether i-eag domains could associate with and regulate hERG R56Q channels in the cellular environment of cardiomyocytes. We report here that hERG R56Q channels heterologously expressed in hiPSC-CMs had faster deactivation kinetics and a less-rectifying I-V relationship and a larger and earlier peak current in response to an AP waveform than that of WT hERG channels, similar to the properties of hERG R56Q expressed in *Xenopus* oocytes or HEK293 cells. The faster deactivation kinetics and I-V relationship and the peak current amplitude and timing of hERG R56Q could be restored to levels similar to that of WT hERG by co-expresssion with i-eag domains.

We detected FRET between hERG R56Q channels which were tagged with Citrine and i-eag domains which were tagged with CFP, which suggests a direct interaction between these two proteins. Based on the functional restoration of deactivation gating and FRET between i-eag domains and hERG R56Q channels, we propose that the regulatory effect of i-eag domains is due to a direct association with hERG R56Q channels. In a model we previously proposed to explain the rescue of gating properties by i-eag domains[22], hERG R56Q channels exhibit faster deactivation kinetics because critical protein interactions between the eag domain and the channel were disrupted. Soluble i-eag domains physically interact with the channel and substitute for the mutated eag domain, thus restoring WT-like deactivation kinetics. Results here show that, as previously detected in the cellular environment of *Xenopus* oocytes and HEK293 cells[17,22], i-eag domains operate in the cellular milieu of cardiomyocytes. We interpret these results to mean that the eag domain of hERG maintains its regulatory role in the environment of different cells, including cardiac cells.

We assessed the role of eag domains on the excitable properties of hERG R56Q in hiPSC-CMs. We first asked whether cells expressing hERG R56Q channels had a change in the APD. Our result (a prolonged AP in cells with hERG R56Q) is opposite to what might be expected due to the similar peak tail current and larger peak current measured in the I-V relationship for hERG R56Q compared to that of WT hERG (S1 Fig). Recordings from Fig 8 and S1 Fig were from the same cells. However, the hERG R56Q current rapidly decays during repolarization, as we measured with a square (Figs 1, 2, 4, 5 and 6) or AP-shaped (Fig 3) voltage command. It is this reduction in current during repolarization that we propose contributes to the prolonged AP. Our results in cells were similar to that of a computational model of the AP with hERG R56Q, in which the altered kinetics of hERG R56Q reduced currents during repolarization and resulted in prolonged APs[24].

We found that heterologous expression of i-eag domains with hERG1a R56Q increased the outward current relative to the tail current, decreased the decay of the repolarizing current either with a square (Figs 1, 2, 4, 5 and 6) or AP-shaped (Fig 3) voltage command, making the hERG R56Q channel currents more like those of WT-hERG1a. Cells with hERG1a R56Q and i-eag domains had a shorter APD than that of cells with hERG1a R56Q (Fig 8). Together, we interpret these results to mean that i-eag associates directly with hERG1a R56Q channels and increases the relative current during repolarization, which in turn shortens the APD. We interpret this to mean that i-eag domains can regulate hERG R56Q channels in the cellular environment of cardiomyocytes.

## Supporting Information

S1 Fig Current-voltage (I-V) relationship of hERG channels. **A,** Tail currents at -50 mV from Fig 4 were normalized to cell capacitance (pF) and plotted *versus* command voltage. **B,** Currents at the end of each depolarizing pulse were normalized to cell capacitance (pF) and plotted *versus* command voltage.
(TIF)
