# Supplementary figures and images for: Eag Domains Regulate LQT Mutant hERG Channels in Human Induced Pluripotent Stem Cell-Derived Cardiomyocytes
Source: PLoS One. 2015 Apr 29;10(4):e0123951. doi: 10.1371/journal.pone.0123951 (PMC4414485; doi:10.1371/journal.pone.0123951)

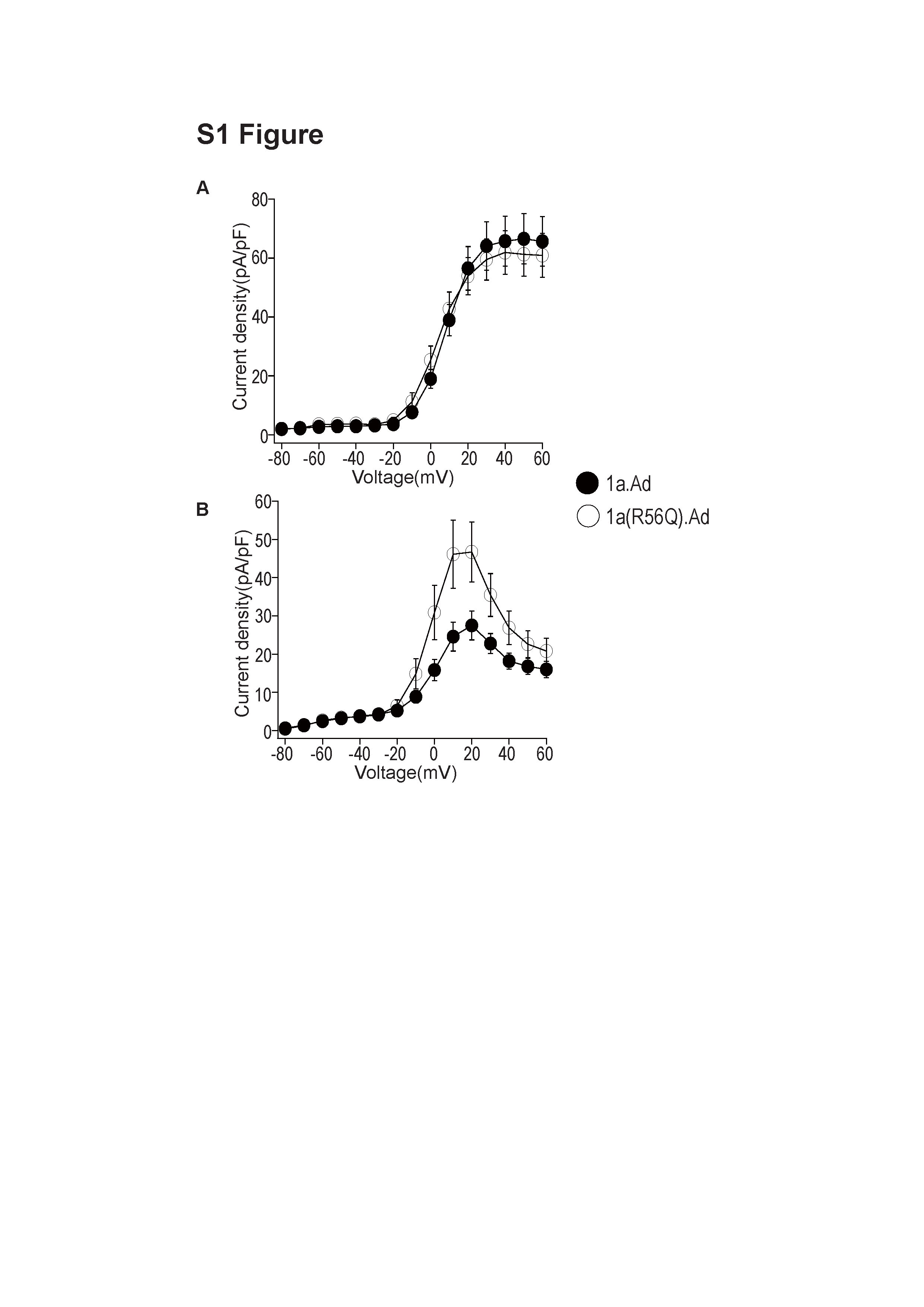

Supplement: S1 Fig — A, Tail currents at -50 mV from Fig 4 were normalized to cell capacitance (pF) and plotted versus command voltage. B, Currents at the end of each depolarizing pulse were normalized to cell capacitance (pF) and plotted versus command voltage. (TIF) [file pone.0123951.s001.tif]
